# Psychological intervention online for adolescent: acceptability of Online Emotional Self-Regulation Improvement program

**DOI:** 10.1192/j.eurpsy.2024.1151

**Published:** 2024-08-27

**Authors:** C. López Soler, A. Martínez, V. Fernandez, M. Castro, J. L. Vicente

**Affiliations:** ^1^Personality, Assesment and psychological treatment; ^2^Medical Psychology; ^3^Evolutionary an Educational Psychology, University of Murcia, Murcia, Spain

## Abstract

**Introduction:**

Given that child and adolescent mental health has been affected by several factors in recent years, such as the distance between home and specialized centers that provide psychological care to children, the lack of care resources, or the lockdown caused by the COVID-19 pandemic, online psychological treatments are becoming increasingly common among the child and adolescent population, although it is necessary to develop this type of treatment for children at psychosocial risk, since these have been developed mostly for the general population. To this aim, the Online Emotional Self-Regulation Improvement program (Mejora de la Auto-regulación para Menores, Online MAM@) was developed.

**Objectives:**

To assess the acceptability of the Online Emotional Self-Regulation Improvement program, by the adolescent.

**Methods:**

The intervention program was applied to a total of n = 32 children (n = 17 girls) between 11 and 15 years of age. The program consists of 7 target emotions to be worked on, and the acceptance, usability, usefulness, enjoyment of each module and barriers to use by the children were assessed with an adaptation of the Venkatesh and Davis scale. These measures were taken post-test, once the intervention module was completed. The program was applied online for five weeks by the children, and their regular therapists contacted them to provide them with weekly access codes and reminders in case they were not completing the modules.

**Results:**

It was observed that the best rated module was the anger module, the most useful module was the sadness module, the module considered to have the highest usability was the fear module, and the most enjoyed module was the sadness module, although all the modules had very high scores above the average and no significant differences were found in the rating of the modules between sexes. As for the most common barriers to use among the children, problems were found with the completion of the intervention, since they often forgot to access the web, and these did not apply what they had learned outside the intervention program.

**Conclusions:**

The Online Emotional Self-Regulation Improvement program is the first program developed in Spanish language for adolescents at psychosocial risk, and may represent a breakthrough to consolidate these programs in the national scene and bring the therapeutic possibilities for adolescents to the same level as in other parts of the world. Focusing on future versions of the program, it would be advisable to reduce its length and incorporate activities outside the treatment program to guarantee a generalization of what is learned in the intervention program in everyday life situations.

**Disclosure of Interest:**

None Declared

